# Bruch’s Membrane Thickness and Retinal Pigment Epithelium Cell Density in Experimental Axial Elongation

**DOI:** 10.1038/s41598-019-43212-8

**Published:** 2019-04-29

**Authors:** Li Dong, Xu Han Shi, Yi Kun Kang, Wen Bin Wei, Ya Xing Wang, Xiao Lin Xu, Fei Gao, Jost B. Jonas

**Affiliations:** 1 Beijing Tongren Eye Center, Beijing Key Laboratory of Intraocular Tumor Diagnosis and Treatment, Beijing Ophthalmology & Visual Sciences Key Lab, Beijing Tongren Hospital, Capital Medical University, Beijing, China; 2 Department of Oncology, Beijing Chao-Yang Hospital, Capital Medical University, Beijing, China; 3 Beijing Institute of Ophthalmology, Beijing Tongren Eye Center, Beijing Tongren Hospital, Capital Medical University, Beijing Ophthalmology & Visual Science Key Lab, Beijing, China; 4 Department of Ophthalmology, Medical Faculty Mannheim of the Ruprecht-Karls-University Heidelberg, Mannheim, Germany

**Keywords:** Refractive errors, Retinal diseases

## Abstract

To assess anatomical changes in eyes with progressive myopia, we morphometrically examined the eyes of guinea pigs with lens-induced axial elongation. Starting at an age of 3–4 weeks, guinea pigs in the experimental group (n = 20 animals) developed unilateral lens-induced axial elongation by wearing goggles for 5 weeks compared to a control group of 20 animals without intervention (axial length:8.91 ± 0.08 mm versus 8.74 ± 0.07 mm; *P* < 0.001). Five weeks after baseline, the animals were sacrificed, and the eyes enucleated. As measured histomorphometrically, Bruch’s membrane thickness was not significantly correlated with axial length in either group at the ora serrata (*P* = 0.41), equator (*P* = 0.41), midpoint between equator and posterior pole (MBEPP) (*P* = 0.13) or posterior pole (*P* = 0.89). Retinal pigment epithelium (RPE) cell density decreased with longer axial length at the MBEPP (*P* = 0.04; regression coefficient beta = −0.33) and posterior pole (*P* = 0.01; beta = −0.40). Additionally, the thickness of the retina and sclera decreased with longer axial length at the MBEPP (*P* = 0.01; beta = −0.42 and *P* < 0.001; beta = −0.64, respectively) and posterior pole (*P* < 0.001; beta = −0.51 and *P* < 0.001; beta = −0.45, respectively). Choroidal thickness decreased at the posterior pole (*P* < 0.001; beta = −0.51). Experimental axial elongation was associated with a thinning of the retina, choroid and sclera and a decrease in RPE cell density, most markedly at the posterior pole. Bruch’s membrane thickness was not related to axial elongation.

## Introduction

Myopia has emerged as a major global public health issue, in particular in East Asia. It has been estimated that by 2050, 49.8% of the world population (4758 million people) will be myopic and that 19.7% of them will be highly myopic (938 million people)^1^. Since myopia, in particular high myopia, is a risk factor for other ocular disorders such as myopic macular degeneration, rhegmatogenous retinal detachment and myopic glaucomatous optic neuropathy, it has become one of the leading causes of blindness and visual impairment^2^.

Axial myopia is associated with a mostly sagittal elongation of the eye, while the enlargement of the globe in its horizontal and vertical dimensions accounts for only about 20% or less of the amount of the axial elongation^3^. It leads to a prolate change in the shape of eyes with progressive axial myopia. Previous studies with humans and animals, such as chicks, guinea pigs, tree shrews, marmosets and macaques, have morphometrically assessed changes taking place in the sclera and choroid of eyes with axial elongation^3–9^. These investigations revealed that the thickness of the sclera progressively decreased with longer axial length. The scleral thinning was most marked at the posterior pole and least marked at the ora serrata and the pars plana region^3,4^. With the thinning of the sclera, the choroidal thickness in particular at the posterior pole also decreased with axial elongation. The reduction in tissue thickness expressed in relative terms was more marked for the choroid than for the sclera. In humans, the choroidal thickness in the subfoveal region decreased from approximately 250 µm in emmetropic eyes to less than 10 µm in highly myopic eyes with myopic maculopathy^5,6^. This change in thickness indicated a reduction by more than 95%. In a similar manner, highly myopic without maculopathy also showed a profoundly decreased choroidal thickness with longer axial length^5,6,10^.

While the thickness of the sclera and choroid have been measured in previous investigations, the thickness of Bruch’s membrane (BM) and the density of retinal pigment epithelium (RPE) cells have not been intensively investigated in experimental studies yet^11,12^. A recent study demonstrated that the biomechanical strength of BM per unit of thickness was up to 100 times higher than the biomechanical strength of the sclera and that the BM could sustain an intraocular pressure of 82 mmHg or higher before rupture^13^. These results suggested that besides the sclera, BM also might play a role in giving form and shape to the eye globe^14^. In view of the biomechanical strength of BM, and if one assumes that BM may be involved in the process of emmetropization and the development of myopia, one would also be interested in the morphometric measures of BM and of the RPE since BM as the basal membrane of the RPE is produced by the RPE. We therefore conducted this study to measure the thickness of BM and the density of RPE cells in guinea pigs.

## Methods

The Ethics Committee of the Beijing Tongren Hospital approved the study and the animal care was conducted according to the ARVO (Association for Vision and Eye Research) Statement for the use of animal in ophthalmic and vision research. Forty healthy pigmented guinea pigs (cavia porcellus) aged 3–4 weeks and weighing 150–200 g were randomly divided into a myopia group (experimental group) (n = 20) and a control group (n = 20). The animals in the experimental group developed a unilateral lens-induced axial elongation by wearing goggles of a refractive power of −12.00 diopters (diameter: 15 mm, optical zone: 12 mm) in front of their right eyes for 5 weeks. The animals in the control group wore goggles with no refractive power in front of their right eyes for 5 weeks. Only one eye per animal was included in the study: the right eye with lens-induced axial elongation in the experimental group and the right eye of the animals of the control group. Using an adhesive tape, the goggles were attached to the skin that had been cleaned and shaved before. Care was taken that the guinea pigs could open the eye and blink freely under the goggles. The goggles were examined daily to ensure they were clean and in place. When needed, they were detached, cleaned and reattached. All guinea pigs were kept at a constant temperature of 26 °C. The light/dark cycle was 12 hours (automatically changed at 8 am and 8 pm) with a luminous intensity of 500 Lux. Food and water were regularly supplied.

Using ultrasonography (A/B-mode scan, oscillator frequency: 11 MHz, Quantel Co., Les Ulis, France), we measured the axial length of all animals at baseline and after five weeks. The sound conducting velocity was assumed to be 1557 m/s for the measurement of the anterior chamber, 1723 m/s for the measurement of the lens and 1540 m/s for the measurement of vitreous chamber^15^. For the axial length measurements, we removed the goggles and administered a topical anesthesia to the cornea (0.5% proparacaine hydrochloride; Alcon, Fort Worth, Tx, USA). The sonographic measurements were repeated until a series of 10 consistent ultrasound measurements had been obtained, the standard deviation of which had to be lower than 0.1 mm. We averaged the 10 measurements and took the mean for further statistical analysis.

**Figure 1:**
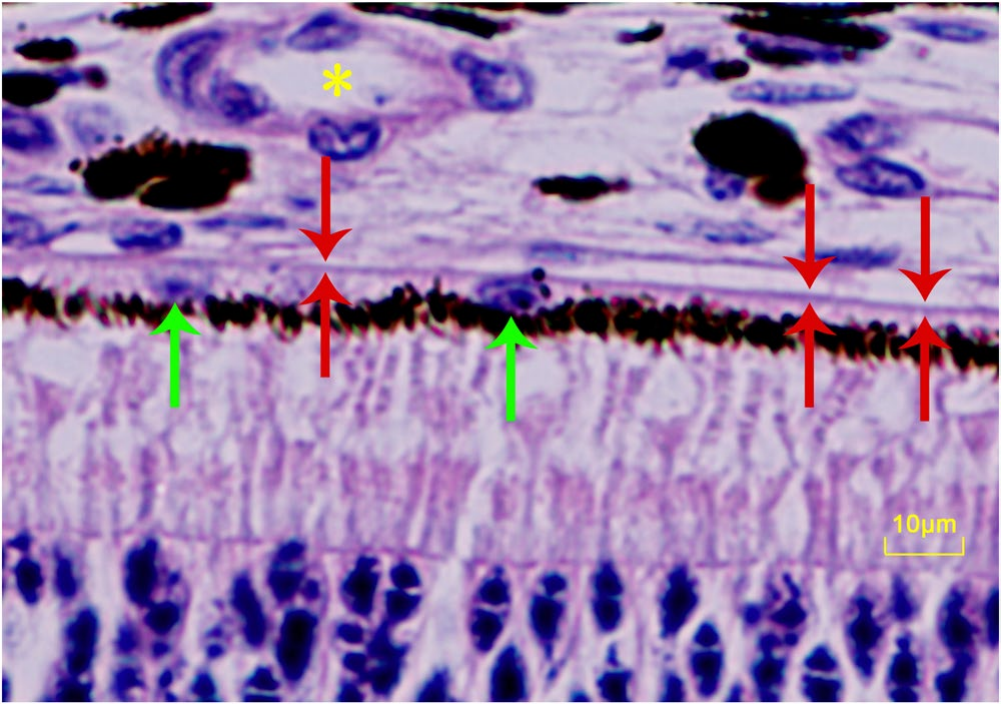
Histopathological photo showing Bruch’s membrane and retinal pigmented epithelium cells. Red arrows: Bruch’s membrane; Green arrows: retinal pigmented epithelium cells; Yellow asterisk: Large choroidal vessel. Hematoxylin and eosin staining.

Five weeks after the baseline, the guinea pigs were sacrificed by an intraperitoneal injection of an overdose of phenobarbital sodium. We enucleated the right eyes after we had marked the 12 o’clock position. We fixed the globes in a solution of 4% formaldehyde for 48 hours. Applying routine procedures, paraffin-embedded horizontal sections of a thickness of 8 µm were prepared and stained by hematoxylin and eosin for the histomorphometrical examination under light microscopy (Olympus Co., Tokyo, Japan). Using a digitized image analysis system, Image-Pro Plus (Media Cybernetics Inc., Rockville, MD, USA), we determined the thickness of BM, retina, choroid and sclera, and the cell density of the RPE per 480 μm length of BM at the ora serrata (the retinal thickness at the ora serrata was measured just posterior to the ora serrata), the equator, the midpoint between the equator and the posterior pole (MBEPP), and at the posterior pole (Fig. 1). During the histomorphometric assessment, the examiner was masked with respect to the group of animals the eye belonged to and with respect to any other data. At each measurement location, we performed 5 measurements of each parameter and took the mean of the five measurements for further statistical analysis.

The statistical analysis was performed using a commercially available software program (SPSS 25.0; IBM, Chicago, IL, USA). The values of the measurements are presented as mean ± standard deviation. The statistical significance of differences between the experimental group and the control group was calculated using Student’s t-tests for independent samples. The statistical significance of differences in the measurements between ocular regions was assessed applying Student’s t-test for paired samples. The ocular regions were compared using a one-way analysis of variance (ANOVA). Finally, we performed a linear regression analysis to detect associations between the outcome parameters (i.e., thickness of BM and retina) and axial length. We calculated the standardized regression coefficient beta, and the non-standardized regression coefficient B including its 95% confidence interval (CI). All *P*-values were 2-sided and were considered statistically significant when <0.05.

The reproducibility of the measurements was assessed by randomly selecting 10 slides and measuring them 10 times in a masked manner at different occasions. The coefficient of variation was calculated as the ratio of the mean of the standard deviations divided by the mean of the means.

## Results

The study included 40 eyes from 40 guinea pigs. The axial length was significantly longer in the experimental group than in the control group (8.91 ± 0.08 mm versus 8.74 ± 0.07 mm; *P* < 0.001) (Table 1). The inter-group difference of 170 µm in axial length would roughly be the equivalent of 5.7 diopters, taking into account the results of the study by Howlett and McFadden. In the latter study, a calculated model predicted that a myopic change in refractive error by one diopter required an axial elongation of about 30 µm for guinea pigs at an age of about 8 weeks^16^.

**Table 1:** Biometric parameters (mean ± standard deviation) of guinea pig eyes.

Parameter	Control group (n = 20)	Experimental group (n = 20)	*P*-value
**Baseline**			
Axial length (mm)	8.10 ± 0.11	8.13 ± 0.08	0.325
Anterior chamber depth (mm)	1.20 ± 0.02	1.21 ± 0.02	0.254
Lens thickness (mm)	3.41 ± 0.03	3.40 ± 0.04	0.416
Vitreous cavity length (mm)	3.49 ± 0.07	3.52 ± 0.05	0.136
**Study end**			
Axial length (mm)	8.74 ± 0.07	8.91 ± 0.08	<0.001
Anterior chamber depth (mm)	1.27 ± 0.03	1.30 ± 0.06	0.026
Lens thickness (mm)	3.75 ± 0.07	3.84 ± 0.07	0.001
Vitreous cavity length (mm)	3.72 ± 0.05	3.77 ± 0.06	0.010

In the total study population, BM was significantly (*P* < 0.001) thicker at the ora serrata, followed by the MBEPP, the posterior pole and the equator. The thickness of BM did not differ significantly (all *P* > 0.10) between the myopia group and the control group at any location of measurement (Table 2).

**Table 2:** Histomorphometrical measurements (mean ± standard deviation) in enucleated guinea pig eyes.

Parameter	Control group	Experimental group	*P*-value
Number	20	20	
**Bruch’s membrane thickness (μm) at:**			
Ora serrata	1.47 ± 0.13	1.47 ± 0.11	0.96
Equator	1.25 ± 0.19	1.18 ± 0.10	0.15
Midpoint between equator and posterior pole (MBEPP)	1.33 ± 0.14	1.36 ± 0.07	0.49
Posterior pole	1.32 ± 0.17	1.26 ± 0.10	0.14
**Retinal pigment epithelium cell density (cells/480 μm) at:**			
Ora serrata	15.2 ± 2.5	14.6 ± 2.0	0.37
Equator	17.7 ± 2.5	16.0 ± 2.5	0.04
MBEPP	19.3 ± 2.7	17.3 ± 2.4	0.02
Posterior pole	20.1 ± 2.7	18.9 ± 2.1	0.06
**Retina thickness (μm) at:**			
Ora serrata^a^	55.2 ± 3.6	54.7 ± 4.2	0.69
Equator	57.2 ± 4.7	55.8 ± 3.6	0.28
MBEPP	84.1 ± 4.6	79.6 ± 6.1	0.01
Posterior pole	98.0 ± 6.6	93.2 ± 7.7	0.04
**Choroidal thickness (μm) at:**			
Ora serrata	22.3 ± 2.4	20.3 ± 2.8	0.02
Equator	24.6 ± 4.6	23.1 ± 4.3	0.31
MBEPP	27.6 ± 4.6	26.0 ± 3.3	0.24
Posterior pole	29.1 ± 2.5	25.5 ± 2.3	< 0.001
**Scleral thickness (μm) at:**			
Ora serrata	80.3 ± 8.5	78.9 ± 13.8	0.70
Equator	64.6 ± 9.2	64.1 ± 6.8	0.83
MBEPP	87.0 ± 5.5	70.9 ± 9.7	<0.001
Posterior pole	89.8 ± 6.3	75.3 ± 13.7	<0.001

^a^Retinal thickness at the ora serrata was measured approximately 250 µm posterior to the ora serrata.

In the total study population, the RPE cell density was the highest at the posterior pole, followed by the MBEPP, the equator and the ora serrata (Table 2). The RPE cell density was higher in the control group than in the experimental group at the equator (*P* = 0.04) and at the MBEPP (*P* = 0.02) (Table 2), while at the posterior pole (*P* = 0.06) and at the ora serrata (*P* = 0.37), the differences between both groups did not reach the level of statistical significance (Table 2).

In both groups, the retina was thickest at the posterior pole (*P* < 0.001), followed by the MBEPP, the equator and the ora serrata (Table 2). The retina was thicker in the control group than in the experimental group when measured at the posterior pole (*P* = 0.04) and at the MBEPP (*P* = 0.01), while the groups did not differ in retinal thickness at the equator (*P* = 0.28) and at the ora serrata (*P* = 0.69).

The choroid was thicker (*P* < 0.001) at the posterior pole and at the MBEPP than at the equator, where it was thicker (*P* = 0.045) than at the ora serrata (Table 2). The choroid was thicker in the control group than in the experimental group at the posterior pole (*P* < 0.001) and at the ora serrata (*P* = 0.02).

The sclera was thicker (*P* = 0.005) at the posterior pole than at the MBEPP, where its thickness did not differ significantly (*P* = 0.78) from the scleral thickness at the ora serrata, where it was significantly (*P* < 0.001) thicker than at the equator (Table 2). The sclera was thicker in the control group than in the experimental group at the posterior pole (*P* < 0.001) and at the MBEPP (*P* < 0.001) (Table 2).

**Figure 2:**
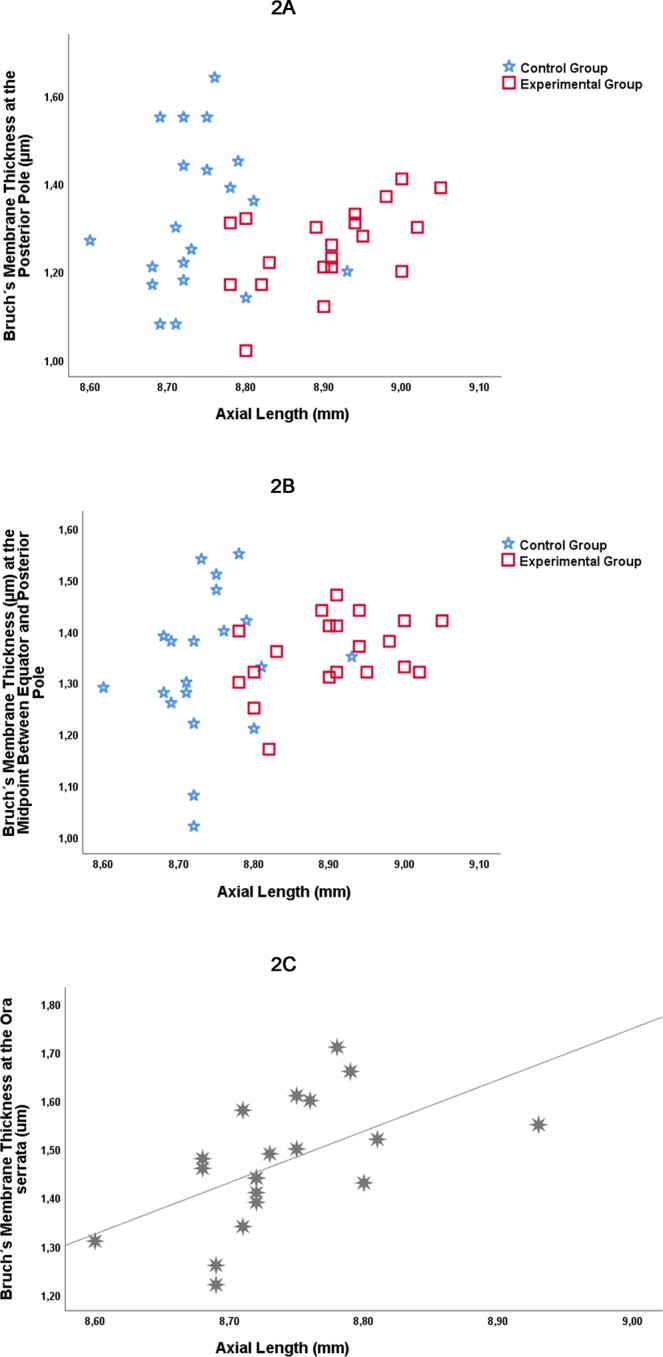
Graphs showing the distribution of Bruch’s membrane thickness by axial length in the experimental group with lens-induced myopia and in the control group at the posterior pole (**A**) and at the midpoint between equator and posterior pole in the experimental group and control group (**B**). (**C**) shows the association between axial length and Bruch’s membrane thickness measured at the ora serrata in the control group.

In the entire study population, the statistical analysis did not reveal any significant associations between BM thickness and axial length, for the BM thickness measurements obtained at the posterior pole (*P* = 0.89) (Fig. 2A), the MBEPP (*P* = 0.13) (Fig. 2B), the equator (*P* = 0.41), or at the ora serrata (*P* = 0.41) (Table 3). For the experimental group alone, BM thickness as measured at the posterior pole increased significantly with longer axial length (beta: 0.54; *P* = 0.01). In the control group, BM thickness measured at the ora serrata increased with longer axial length (beta: 0.55; *P* = 0.01) (Fig. 2C). At all other measurement points in both groups, BM thickness was not significantly correlated with axial length.

**Table 3:** Histomorphometrical measurements in association with axial length in enucleated guinea pig eyes.

Region	Mean ± SD	Median	Range	Association with axial length				
				Nonstandardized coefficient B	Standardized coefficient beta	*P*-Value	95% Confidence Interval of B	Equation
**Bruch’s membrane thickness at: (μm)**								
Ora serrata	1.47 ± 0.12	1.48	1.22–1.73	0.13	0.13	0.41	−0.18, 0.43	
Equator	1.22 ± 0.16	1.21	0.92–1.62	−0.10	−0.13	0.41	−0.33, 0.14	
Midpoint between equator and posterior pole (MBEPP)	1.35 ± 0.11	1.36	1.02–1.55	0.25	0.24	0.13	−0.08, 0.58	
Posterior pole	1.29 ± 0.14	1.28	1.02–1.64	−0.02	−0.02	0.89	−0.28, 0.25	
**Retinal pigment epithelium cell density (cells/480 μm) at:**								
Ora serrata	14.9 ± 2.2	15	11–19	−0.02	−0.30	0.06	−0.03, 0.00	
Equator	16.8 ± 2.6	17	12–21	−0.01	−0.31	0.05	−0.03, 0.00	
MBEPP	18.3 ± 2.7	18	13–24	−0.01	−0.33	0.04	−0.03, −0.00	Y = −7.9 × + 88.0
Posterior pole	19.3 ± 2.5	19	14–24	−0.02	−0.40	0.01	−0.03, −0.00	Y = −8.8 × + 96.6
**Retina thickness at: (μm)**								
Ora serrata^a^	54.9 ± 3.9	55	45–62	0.00	0.02	0.90	−0.01, 0.01	
Equator	56.5 ± 4.1	56	49–67	−0.00	−0.02	0.90	−0.01, 0.01	
MBEPP	81.8 ± 5.8	83	70–92	−0.01	−0.42	0.01	−0.01, −0.00	Y = −21.7 × + 273.2
Posterior pole	95.6 ± 7.5	95	80–109	−0.01	−0.51	<0.001	−0.01, −0.00	Y = −33.8 × + 393.5
**Choroid thickness at: (μm)**								
Ora serrata	21.3 ± 2.8	21	17–28	−0.01	0.27	0.10	−0.02, 0.00	
Equator	23.8 ± 4.5	23	20–39	−0.00	−0.06	0.73	−0.01, 0.01	
MBEPP	26.8 ± 4.0	27	21–37	−0.00	−0.10	0.53	−0.01, 0.01	
Posterior pole	27.3 ± 3.0	27	22–34	−0.02	−0.51	0.00	−0.03, −0.01	Y = −13.3 × + 144.8
**Sclera thickness at: (μm)**								
Ora serrata	79.6 ± 11.4	76	69–119	0.00	0.08	0.61	−0.00, 0.00	
Equator	64.4 ± 8.0	65	51–77	0.00	0.19	0.24	−0.00, 0.01	
MBEPP	79.0 ± 11.2	81	58–98	−0.01	−0.64	<0.001	−0.01, −0.00	Y = −64.0 × + 643.8
Posterior pole	82.5 ± 12.8	86	58–101	−0.00	−0.45	<0.001	−0.01, −0.00	Y = −51.4 × + 536.3

^a^Retinal thickness at the ora serrata was measured approximately 250 µm posterior to the ora serrate.

**Figure 3:**
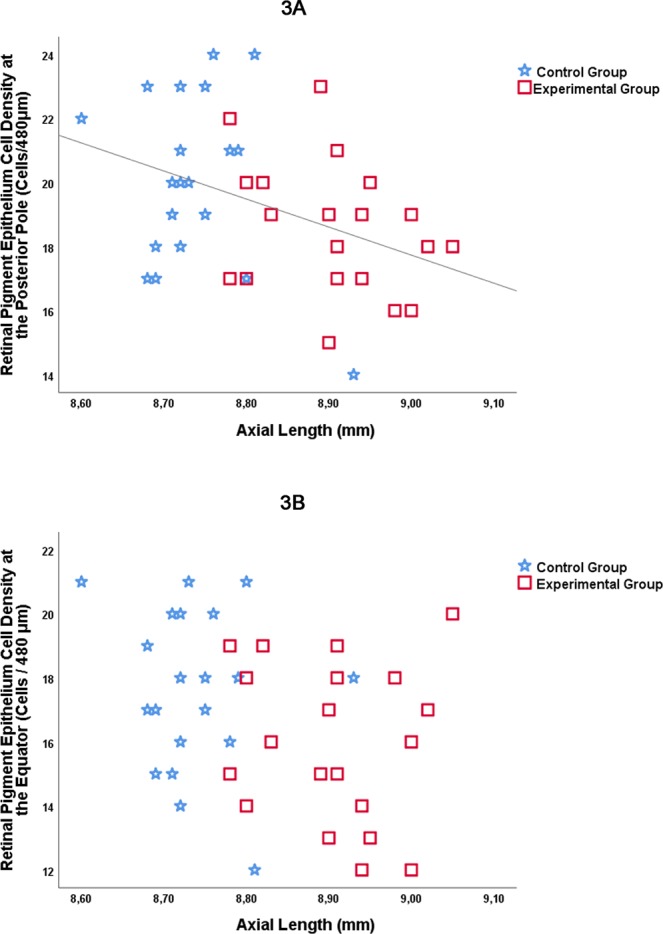
Graphs showing the distribution of the retinal pigment epithelium cell density (cells/480 μm) by axial length at the posterior pole (**A**) and at the equator (**B**) in young guinea pigs without intervention (control group) and in guinea pigs with lens-induced myopia (experimental group). The equation for the regression line in (**A**) was: Retinal Pigmented Epithelium Cell Density (Cells/480 μm) = −9.6 × Axial Length (mm) + 97.

Taking the entire study population, the RPE cell density measured at the posterior pole (*P* = 0.01; beta = −0.40) (Fig. 3A) and at the MBEPP (*P* = 0.04; beta = −0.33) decreased significantly with longer axial length, while the RPE cell density at the equator (*P* = 0.05; beta: −0.31) (Fig. 3B) and at the ora serrata (*P* = 0.06; beta: −0.30) was only marginally significantly correlated with longer axial length (Table 3) (Fig. 3). Stratifying the study population into the experimental group and control group revealed similar trends for a decline of the RPE densities with increasing axial length. The associations however did not reach the level of statistical significance (experimental group, RPE density at posterior pole: *P* = 0.21; beta = −0.29; at MBEPP: *P* = 0.37; beta = −0.21; at equator: *P* = 0.72; beta: −0.09; at ora serrata: *P* = 0.12; beta: −0.36; control group: RPE density at posterior pole: *P* = 0.27; beta = −0.26; at MBEPP: *P* = 0.75; beta = 0.08; at equator: *P* = 0.55; beta: −0.14; at ora serrata: *P* = 0.30; beta: −0.25).

The thickness of the retina decreased significantly with longer axial length for the measurements taken at the posterior pole (*P* < 0.001, beta = −0.51) and at the MBEPP (*P* = 0.01, beta = −0.42) (Table 3). Stratifying the study population into the experimental group and the control group showed that the association between a thinner retina and longer axial length was significant for the measurement at the posterior pole in the control group (*P* < 0.001; beta: −0.80), while at all other measurement points in both groups, the association was not statistically significant (*P* ≥ 0.20)

The thickness of the sclera decreased significantly with longer axial length for the measurements taken at the posterior pole (*P* < 0.001, beta = −0.45) and at the MBEPP (*P* < 0.001, beta = −0.64) (Table 3). Stratifying the study population into the experimental group and the control group revealed that the association between a thinner sclera and longer axial length was statistically significant only for the measurement at the midpoint in the experimental group (*P* = 0.04; beta: −0.46).

The thickness of the choroid decreased significantly with longer axial length for measurements obtained at the posterior pole (*P* < 0.001; beta = −0.51), while no significant correlations were found between axial length and choroidal thickness measured at the ora serrata, the equator or the MBEPP (all *P* > 0.05) (Table 3). Within the experimental group and the control group, none of the associations reached the level of statistical significance (*P* > 0.05).

The coefficient of variation for re-determinations of the thickness of BM, retina, choroid and sclera and for re-counting the RPE cells were 9.8%, 8.3%, 6.9%, and 7.0% at the equator, and 9.2%, 7.3%, 8.7%, and 8.0% at the posterior pole, respectively.

## Discussion

Our study on experimental myopia in young guinea pigs showed that the thickness of BM, in contrast to the thickness of the retina, choroid and sclera and in contrast to the RPE cell density, did not decrease with longer axial length. The thickness of BM was statistically independent of axial length, or it showed a tendency towards higher thickness measurements with longer axial length for measurements taken at the posterior pole in the experimental group and for measurements taken at the ora serrata in the control group. Since longer ocular axial length leads to a larger inner ocular surface area, the finding of BM thickness being mostly independent of axial length indicates an increase in the volume of BM with longer axial length.

Except for the aforementioned studies on human enucleated globes, other investigations have not yet been focused on the relationship between BM thickness and axial length. The BM was thicker in the human eyes than in the guinea pig eyes^11,12^. To cite an example, the mean BM thickness at the posterior pole was 2.0 ± 0.4 µm in the human eyes versus 1.32 ± 0.17 µm in the guinea pigs eyes of the present study. In the human eyes as well as in the guinea pig eyes, BM thickness was not significantly correlated with axial length. An exception were human eyes with congenital glaucoma and secondary high myopia in which BM was thinner than in human eyes with primary high myopia or in human eyes with normal axial length and in which BM thickness decreased with longer axial length^11,12,17^.

The finding that BM thickness did not decrease with axial length suggests that the volume of BM increased since the inner surface area of the eyes enlarged. The notion of an enlarging BM in the axially elongating eyes fits with the finding that the density of the RPE decreased with longer axial length (assuming that the RPE cells did not multiply). The RPE cell density decreased at all measurement locations, and it was statistically significant at the MBEPP and at the posterior pole (Table 3) (Fig. 3A,B). It was in contrast to human eyes, in which the RPE cell density decreased with longer axial length at the equator and at the MBEPP but not at the posterior pole^18,19^. The reason for this discrepancy between guinea pig eyes and human globes may be differences in the physiology and anatomy of the eye, in particular in the morphology of the posterior pole^20–23^. Human eyes, in contrast to guinea pig eyes, have a fovea, while the guinea pig eye has a visual streak with an increased retinal ganglion cell density in the horizontal meridian^22^. Studies on the foveal region of human eyes revealed that the thickness of the retina, the RPE cell density, the length of BM and best corrected visual acuity were independent of axial length^24–27^. It was assumed that in human eyes the fundus periphery guides the process of emmetropization^28–32^. Axial elongation serving the process of emmetropization would be achieved by the expansion of BM predominantly in the equatorial and pre-equatorial region^18^. It would explain the axial elongation-associated decrease in RPE cell density and retinal thickness at the equator and at the MBEPP in human eyes^19^. It would also fit with the observation that the foveal structures seemed to be spared with axial elongation. In guinea pig eyes without a fovea, the enlargement of BM may take place in a more diffuse manner and may include the posterior pole. This notion is supported by the findings obtained in the present study.

We detected that BM thickness in the guinea pig eyes was thickest at the ora serrata, followed by the MBEPP, the posterior pole and the equator. The RPE cell density was highest at the posterior pole, followed by the MBEPP, the equator and the ora serrata. These findings were consistent with observations made in previous studies on human eyes^11,12,33^. In an investigation on normal human donor eyes, the RPE cell density decreased significantly (*P* < 0.001) from the fovea (4,220 ± 727 cells/mm^2^) to the midperiphery (3,002 ± 460 cells/mm^2^) and to the outer peripheral fundus regions (1,600 ± 411 cells/mm^2^)^33^.

As in human eyes enucleated for clinical reasons, the eyes of the guinea pigs in the present study showed an axial length-associated reduction in scleral thickness at the posterior pole and at the MBEPP, while the scleral thickness at the equator and at the ora serrata was not significantly correlated with axial length^9^. Parallel to the thinning of the sclera, the choroidal thickness at the posterior pole decreased with axial length in the present study, while the choroidal thickness at the other measurement locations was not significantly correlated with axial length (Table 3). It suggests that in guinea pigs, as in human eyes, axial elongation has a major effect at the posterior pole.

Limitations of our study should be discussed. BM thickness with values ranging between 1 and 2 µm were relatively close to the spatial resolution of conventional light microscopy so that it could have been difficult to precisely measure BM thickness in some eyes in the present study. The assessment of the reproducibility of the measurements revealed however a relatively low coefficient or variation (<10%) for remeasurements of BM thickness. It has remained unclear whether and how far results of the present experimental study can be transferred to clinical situations in human eyes. The range in axial length in the study population was relatively small, such that the lack of a statistically significant association between BM thickness and axial length might have been due to the small range of axial length. The distribution of the values as demonstrated on the scattergram in Fig. 2A may make it however unlikely that with a larger range of axial length measurements and a larger number of animals included in the study of the association between BM thickness and axial length might have become statistically significant. Histomorphometry is a relatively imprecise method for measuring choroidal thickness that can be markedly affected by changes in blood flow in the choroid shortly before enucleation, post-enucleation loss of choroidal blood filling, and artifacts due to the preparation of the histological slides including postmortem swelling, shrinkage due to the fixation, and artificial detachment of the choroid from the inner scleral surface. To a slightly lesser degree, the same holds true for the histological measurement of the thickness of the retina and other ocular tissues. Despite these limiting factors of histomorphometry increasing the noise in the determinations, the experimental group and the control group differed significantly in the thickness of the retina, choroid and retina as measured at the posterior pole. It may serve to underscore the conclusion that the axial elongation was associated with a thinning of these tissues at the posterior pole. Techniques to measure the thickness of the retina, choroid and sclera intravitally were unfortunately not available. In a parallel manner, measuring the RPE cell density on histological sagittal sections is less precise than determining the RPE cell density on flat mounts. Factors making the counting of RPE cells on histologic sections difficult include the limited visibility of the RPE cells due to the high concentration of melanin within the cells and differences in cell size. Studies of human eyes showed a marked variability in RPE cell size with the smallest RPE cells located in the foveal center^33^. It was, however, not possible to obtain flat mounts and sagittal sections from the same eyes. While we applied a lens of −12 diopters to induce axial elongation, other investigations on lens-induced axial elongation in guinea pigs used lenses with a lower refractive power, such as −10 diopters and −4 diopters. The values for histological measures in our study were lower than those reported previously in other studies. For example, the maximum central retinal thickness was approximately 140 μm in the study by Buttery and colleagues, while in our study, mean retinal thickness at the posterior pole was 98.0 ± 6.6 μm^22^. Applying optical coherence tomography in adult guinea pigs, Jnawali and colleagues reported on *in-vivo* measurements of the retinal thickness of 147.7 ± 5.8 μm and of the choroidal thickness of 64.8 ± 11.6 μm^34^. The reasons for the discrepancy in the thickness measurements between the studies may have been differences in the age of the animals and in the techniques applied, including the difference between *in-vivo* measurements versus histomorphometrical determinations and histological fixation-induced shrinkage artifacts. It was, however, not the goal of the present study to assess normative measurements of the ocular anatomy of the guinea pig, but to compare the experimental group and control group and to assess associations with axial elongation. Any methodological factor influencing the histomorphometrical values should have had an effect on the whole study population and might not have influenced differences between the experimental group and control group.

In conclusion, in this experimental model, axial elongation was associated with a thinning of the retina, choroid and sclera and a decrease in the RPE cell density, while the thickness of BM was statistically independent of axial length. It suggests an axial elongation-associated increase in the volume of BM.

## References

[1] Holden BA (2016). Global Prevalence of Myopia and High Myopia and Temporal Trends from 2000 through 2050. Ophthalmology..

[2] Morgan IG, Ohno-Matsui K, Saw SM (2012). Myopia. Lancet..

[3] Heine L (1899). Beiträge zur Anatomie des myopischen Auges. Arch. Augenheilk..

[4] Shen L (2015). Scleral thickness in Chinese eyes. Invest. Ophthalmol. Vis. Sci..

[5] Margolis R, Spaide RF (2009). A pilot study of enhanced depth imaging optical coherence tomography of the choroid in normal eyes. Am. J. Ophthalmol..

[6] Wei WB (2013). Subfoveal choroidal thickness: the Beijing Eye Study. Ophthalmology..

[7] McBrien NA, Cornell LM, Gentle A (2001). Structural and ultrastructural changes to the sclera in a mammalian model of high myopia. Invest. Ophthalmol. Vis. Sci..

[8] Wildsoet C, Wallman J (1995). Choroidal and scleral mechanisms of compensation for spectacle lenses in chicks. Vision Res..

[9] Vurgese S, Panda-Jonas S, Jonas JB (2012). Scleral thickness in human eyes. PLoS One..

[10] Burfield HJ, Patel NB, Ostrin LA (2018). Ocular biometric diurnal rhythms in emmetropic and myopic adults. Invest. Ophthalmol. Vis. Sci..

[11] Jonas JB, Holbach L, Panda-Jonas S (2014). Bruch’s membrane thickness in high myopia. Acta Ophthalmol..

[12] Bai HX (2017). Bruch’s membrane thickness in relationship to axial length. PLoS One..

[13] Wang X (2018). Biomechanical properties of Bruch’s membrane-choroid complex and their influence on optic nerve head biomechanics. Invest. Ophthalmol. Vis. Sci..

[14] Jonas JB, Holbach L, Panda-Jonas S (2014). Scleral cross section area and volume and axial length. PLoS One..

[15] Lu F (2009). Axial myopia induced by hyperopic defocus in guinea pigs: A detailed assessment on susceptibility and recovery. Exp. Eye Res..

[16] Howlett MH, McFadden SA (2007). Emmetropization and schematic eye models in developing pigmented guinea pigs. Vision Res..

[17] Jonas JB, Holbach L, Panda-Jonas S (2016). Histologic differences between primary high myopia and secondary high myopia due to congenital glaucoma. Acta Ophthalmol..

[18] Jonas JB, Ohno-Matsui K, Jiang WJ, Panda-Jonas S (2017). Bruch membrane and the mechanism of myopization. A new theory. Retina..

[19] Jonas JB, Ohno-Matsui K, Holbach L, Panda-Jonas S (2017). Retinal pigment epithelium cell density in relationship to axial length in human eyes. Acta Ophthalmol..

[20] Ostrin LA, Garciam MB, Choh V, Wildsoet CF (2014). Pharmacologically stimulated pupil and accommodative changes in Guinea pigs. Invest Ophthalmol Vis Sci..

[21] Do-Nascimento JL, Do-Nascimento RS, Damasceno BA, Silveira LC (1991). The neurons of the retinal ganglion cell layer of the guinea pig: quantitative analysis of their distribution and size. Braz. J. Med. Biol. Res..

[22] Buttery RG, Hinrichsen CFL, Weller WL, Haight JR (1991). How thick should a retina be? A comparative study of mammalian species with and without intraretinal vasculature. Vision Res..

[23] Ostrin LA, Wildsoet CF (2016). Optic nerve head and intraocular pressure in the guinea pig eye. Exp. Eye Res..

[24] Jonas JB (2015). Macular Bruch’s membrane length and axial length. The Beijing Eye Study. PloS One..

[25] Jonas JB (2016). Retinal thickness and axial length. Invest. Ophthalmol. Vis. Sci..

[26] Yin G (2012). Ocular axial length and its associations in Chinese. The Beijing Eye Study. PLoS One..

[27] Jonas RA (2015). Optic disc-fovea distance, axial length and parapapillary zones. The Beijing Eye Study 2011. PloS One..

[28] Smith EL (2010). Effects of optical defocus on refractive development in monkeys: evidence for local, regionally selective mechanisms. Invest. Ophthalmol. Vis. Sci..

[29] Huang J, Hung LF, Smith EL (2011). Effects of foveal ablation on the pattern of peripheral refractive errors in normal and form-deprived infant rhesus monkeys (Macaca mulatta). Invest. Ophthalmol. Vis. Sci..

[30] Berntsen DA, Barr CD, Mutti DO, Zadnik K (2013). Peripheral defocus and myopia progression in myopic children randomly assigned to wear single vision and progressive addition lenses. Invest. Ophthalmol. Vis. Sci..

[31] Benavente-Pérez A, Nour A, Troilo D (2014). Axial eye growth and refractive error development can be modified by exposing the peripheral retina to relative myopic or hyperopic defocus. Invest. Ophthalmol. Vis. Sci..

[32] Arumugam B (2016). The effects of the relative strength of simultaneous competing defocus signals on emmetropization in infant rhesus monkeys. Invest. Ophthalmol. Vis. Sci..

[33] Panda-Jonas S, Jonas JB, Jakobczyk-Zmija M (1996). Retinal pigment epithelial cell count, distribution, and correlations in normal human eyes. Am. J. Ophthalmol..

[34] Jnawali A, Beach KM, Ostrin LA (2018). *In vivo* imaging of the retina, choroid, and optic nerve head in guinea pigs. Curr. Eye Res..

